# From Predictors to Mechanisms: Interpretable Artificial Intelligence Evidence on Mathematics Achievement and Cognitive Learning Systems

**DOI:** 10.3390/jintelligence14060091

**Published:** 2026-05-25

**Authors:** Danyang Meng, Alan T. K. Wan

**Affiliations:** 1 Department of Mathematics, East China Normal University, Shanghai 200241, China; 52265500036@stu.ecnu.edu.cn; 2 Department of Decision Analytics and Operations, City University of Hong Kong, Hong Kong, China

**Keywords:** mathematical achievement, learning intelligence, interpretable artificial intelligence, cognitive mechanisms, self-regulated learning, PISA, cross-regional comparison

## Abstract

Understanding academic achievement requires moving beyond the identification of influential factors toward explaining how these factors are organized into functional learning and cognitive mechanisms. Although prior research has extensively documented the roles of socioeconomic status, student attitudes, and learning behaviors, less attention has been paid to how these elements interact within structured pathways that reflect underlying learning intelligence across educational systems. This study adopts a mechanism-oriented perspective to examine mathematics achievement using data from PISA 2018. Focusing on high-performing regions in East Asia and Western countries, it integrates interpretable artificial intelligence methods with structural modeling to investigate how contextual, psychological, and learning-process factors jointly shape achievement outcomes. The findings show that high achievement is not governed by a single set of dominant predictors, but by distinct organizational mechanisms of learning intelligence. In East Asian systems, achievement follows a *chain-like convergent structure*, in which socioeconomic background is systematically translated into academic outcomes through sequential psychological and self-regulatory processes. Psychological factors, particularly educational expectations and self-beliefs, function as key mediating mechanisms that organize learning engagement and strategy use. By contrast, high-performing systems in Europe and North America exhibit a *parallel configuration*, in which multiple cognitive and behavioral factors independently contribute to achievement through more decentralized pathways, reflecting a distributed structure of learning intelligence. Across regions, learning processes such as reading engagement and digital literacy show consistently positive associations with achievement. However, their roles vary depending on how they are embedded within broader system-level structures. These results suggest that self-regulation operates not merely as an associated factor, but as an organizing mechanism of learning intelligence that structures the translation of background resources into performance. By reconceptualizing prediction as a means of revealing the organization of learning intelligence, this study proposes a unified analytical framework that links interpretable artificial intelligence with theory-driven explanation. The findings contribute to a deeper understanding of how achievement systems function and highlight that high performance can emerge through multiple, structurally distinct pathways, with important implications for educational research, cognitive theory, and policy design.

## 1. Introduction

International Large-Scale Assessments (ILSAs), as a central component of contemporary educational measurement, have played an increasingly important role in comparative education research and educational policy analysis. Rutkowski et al. (2014) note that ILSAs are designed to achieve cross-cultural comparability and competence-oriented measurement. Through standardized assessment frameworks and complex sampling techniques, they provide a systematic portrayal of student competencies across diverse educational systems. Beyond generating highly representative and comparable data, ILSAs have also contributed to a fundamental shift in how educational outcomes are conceptualized, from knowledge acquisition to competence-based performance.

Among these international assessment programs, the Programme for International Student Assessment (PISA), developed by the Organisation for Economic Co-operation and Development (OECD), is widely regarded as a paradigmatic example of competence-oriented assessment. Rather than focusing on the mastery of specific curricular content, PISA emphasizes students’ ability to apply knowledge and skills to solve problems in real-world contexts. This shift reflects a broader transformation from the reproduction of knowledge to the demonstration of competencies (OECD, 2019, 2020). Within this framework, the concept of mathematical ability has evolved into the concept of mathematical literacy, defined as the capacity to formulate, employ, and interpret mathematics in a variety of contexts (OECD, 2019). This conceptualization highlights the functional and contextualized nature of knowledge and has been widely adopted as a key indicator of higher-order cognitive competencies (Stacey, 2015).

Building on this perspective, a large body of research has examined the determinants of students’ mathematics achievement, which can be reinterpreted as an empirical foundation for understanding mathematical literacy. Prior studies have consistently identified background characteristics, learning attitudes, and classroom behaviors as key predictors of student performance across different educational stages (Huang, 2016; Y. Zhang, 2020). With the transition toward competence-oriented assessment, these variables have increasingly been incorporated into the explanatory framework of mathematical literacy.

In addition to individual-level determinants, the evaluation of learning outcomes has emerged as another important dimension in understanding high-performing educational systems. Competence-oriented assessment emphasizes the alignment between learning outcomes and educational objectives, positioning evaluation not only as a measurement tool but also as a mechanism for instructional adjustment and quality improvement. Chen (2017) argue that the alignment between outcome evaluation and instructional adaptation can optimize teaching processes and foster competency development. Furthermore, within the context of professional accreditation and quality assurance, outcome-based evaluation has been increasingly institutionalized (Xiao, 2022). These evaluation systems, through indicator design and feedback mechanisms, play a critical role in shaping both teaching practices and student development.

However, despite these advances, existing research remains subject to several important limitations. First, most studies adopt a variable-centered perspective, focusing on identifying significant predictors of achievement while paying limited attention to how these factors are structurally organized into coherent mechanisms. Second, although cross-national comparisons are widely conducted using ILSA data, relatively few studies systematically examine how the mechanisms underlying mathematical literacy differ across regions. Third, methodological approaches are still dominated by traditional statistical models, which may be limited in their ability to capture complex, nonlinear interactions among variables.

In recent years, machine learning has emerged as a promising methodological alternative for educational data analysis. Compared with traditional statistical approaches, machine learning techniques offer enhanced capabilities in handling high-dimensional data, modeling nonlinear relationships, and identifying complex interaction patterns. Evidence from the field of test security suggests that machine learning methods, including supervised, unsupervised, and semi-supervised learning, can outperform conventional statistical approaches in detecting abnormal behaviors and potential risks (Gao & Li, 2024). Nevertheless, many machine learning models operate as “black boxes,” providing limited interpretability and thereby constraining their usefulness for theory building and mechanism explanation.

To address these gaps, this study proposes a mechanism-oriented analytical framework based on interpretable machine learning. By integrating predictive modeling with interpretability techniques, the study aims not only to identify key predictors of mathematical literacy but also to reveal how these factors are organized into functional mechanisms across different educational systems.

Specifically, using PISA 2018 data, this study employs interpretable machine learning methods such as CatBoost and SHAP to examine cross-regional differences in mathematical literacy. The analysis focuses on high-performing regions, with the goal of uncovering both shared patterns and region-specific mechanisms. The findings indicate that, while similar sets of predictors are identified across regions, their structural organization and relative importance differ substantially, suggesting distinct pathways through which mathematical literacy is developed.

This study develops a mechanism-oriented comparative framework for examining how contextual conditions, psychological organization, and learning processes jointly shape mathematical literacy across high-performing educational systems. By integrating interpretable machine learning and structural pathway analysis, the study investigates whether similar levels of achievement may emerge through structurally distinct learning-system organizations. The following section develops the integrated theoretical framework and corresponding analytical expectations that guide the empirical analysis.

The present study should be interpreted as a comparative analytical investigation of learning-system organization based on PISA 2018, rather than as a causal or temporally predictive account of contemporary educational systems. Because the analyses rely on cross-sectional large-scale assessment data collected prior to major post-2020 educational transformations, the findings primarily reflect structural patterns associated with the educational contexts represented in PISA 2018.

## 2. Literature Review

### 2.1. Conceptualization and Framework of Mathematical Literacy

With the increasing societal demand for scientific literacy and quantitative reasoning, research in mathematics education has gradually shifted from a sole focus on the acquisition of mathematical knowledge to an emphasis on individuals’ ability to understand and apply mathematics in real-world contexts. Within this context, the concept of mathematical literacy has emerged as a central construct for describing students’ learning outcomes in mathematics.

Compared with traditional achievement-oriented evaluations that prioritize the mastery of curricular knowledge, mathematical literacy places greater emphasis on individuals’ capacity to use mathematics for analysis, reasoning, and decision-making in everyday life, social participation, and problem-solving situations (Steen, 2001).

Mathematical literacy has been conceptualized from multiple perspectives in the existing literature, with particular emphasis on its significance for educational practice and real-world application. One influential perspective views mathematical literacy as a broader educational vision that extends beyond traditional mathematics instruction, highlighting students’ ability to use mathematical ideas and skills to understand and respond to diverse real-world situations.

Within this framework, mathematical literacy encompasses not only classroom learning content but also a range of pedagogical strategies. These include the use of authentic and contextualized problems, the integration of technological tools to support learning, and the design of assessment approaches that reflect real-world quantitative relationships. Such practices aim to equip students with competencies that remain applicable and transferable throughout adulthood (Burkhardt et al., 2024).

Building on this perspective, some studies have further provided systematic accounts of mathematical literacy from a structural modeling perspective. For example, mathematical literacy has been conceptualized as comprising four core components—knowledge, application, disposition, and learning—which together capture both cognitive and behavioral dimensions of students’ mathematical engagement (Denischeva et al., 2021; Tso & Lei, 2018).

These components are often linked through a “learning loop,” in which real-world problems are transformed into mathematical representations, and the resulting solutions are subsequently interpreted within the original context. This iterative process reflects the dynamic nature of students’ cognitive development and highlights the process-oriented characteristics of mathematical literacy.

Such a cyclical structure not only provides insight into how mathematical literacy develops over time, but also offers an important foundation for designing curriculum materials and instructional approaches aligned with a mathematical literacy perspective (Denischeva et al., 2021; Tso & Lei, 2018).

Aligned with this shift toward a competence-oriented perspective, PISA has progressively developed an integrated assessment framework that links *context*, *content*, *process*, and *competency*. Within this framework, the process dimensions of *formulate*, *employ*, and *interpret* are used to systematically capture students’ cognitive progression from problem understanding and model construction to solution interpretation and reflection.

Stacey (2011) argue that this process-oriented structure helps move beyond an assessment logic centered solely on the correctness of final answers by bringing students’ reasoning pathways and modeling processes into the focus of evaluation. Compared with traditional classroom practices that emphasize procedural computation and fixed problem types, this framework highlights the processual, contextualized, and open-ended nature of mathematical learning.

Consequently, it promotes a fundamental shift in assessment orientation—from an answer-focused paradigm toward one that prioritizes processes and competencies (Alagumalai & Buchdahl, 2021).

### 2.2. Determinants of Students’ Mathematical Literacy

The determinants of students’ mathematical literacy can be understood within a multi-level framework encompassing individual characteristics, family environments, school practices, and broader socio-cultural contexts. Existing research suggests that these factors do not operate independently, but interact in complex and often nonlinear ways to shape students’ competency development.

At the individual level, a substantial body of research has examined cognitive and affective factors associated with mathematical literacy. Evidence regarding gender differences remains inconclusive. For example, studies on lower secondary students have found no significant differences between male and female students in mathematical literacy, a finding partly attributed to the limited availability of literacy-oriented problem contexts in classroom instruction (Qirom et al., 2023). Beyond demographic characteristics, self-regulated learning (SRL) has emerged as a key individual mechanism. SRL refers to students’ capacity to monitor and regulate their cognition, motivation, and behavior throughout the learning process. Students with stronger self-regulatory capabilities tend to demonstrate higher levels of mathematical literacy, as these competencies enable them to sustain effort in complex problem-solving situations and maintain confidence in their reasoning processes (Gabriel et al., 2020). This suggests that individual-level factors may influence outcomes not merely in a direct manner, but through regulatory mechanisms that organize learning processes.

At the family level, the home learning environment plays a crucial role in shaping early mathematical development. It typically includes parental attitudes toward mathematics, the frequency of home-based learning activities, and the availability of educational resources. Empirical evidence indicates that positive parental attitudes and engagement in mathematics-related activities are significantly associated with children’s arithmetic fluency. However, other factors, such as parental assistance with homework or parental mathematics anxiety, do not consistently exhibit direct effects on achievement (Skwarchuk et al., 2022). This differentiated pattern suggests that family influences may operate through selective pathways rather than uniform direct effects, highlighting the importance of understanding how specific aspects of the home environment are translated into learning processes.

At the school level, instructional practices constitute another major source of variation in students’ mathematical literacy. Research shows that practices such as cognitive activation, exposure to pure mathematical tasks, and participation in extracurricular mathematics activities are positively associated with students’ performance (H. Zhang, 2018). At the same time, certain pedagogical approaches, such as excessive reliance on student-centered instruction or the overuse of formative assessment, have been found to produce negative effects. These findings indicate that instructional practices do not uniformly support learning outcomes, and that their effects depend on how they are enacted and integrated within broader teaching systems. In this sense, school-level influences reflect complex instructional mechanisms rather than simple input–output relationships.

At the socio-cultural level, mathematical literacy is embedded within broader cultural and social contexts. Students’ experiences outside formal schooling shape their approaches to reasoning and problem solving. Studies involving immigrant learners, for example, show that students construct mathematically meaningful representations through real-life decision-making processes, demonstrating the close connection between mathematical understanding and social contexts (Croce, 2020). This perspective highlights that mathematical literacy is not solely an individual cognitive outcome, but also a context-dependent and socially constructed competence. Consequently, standardized assessment instruments may not fully capture these contextual influences.

Taken together, while existing research has identified a wide range of influential factors across individual, family, school, and socio-cultural levels, these factors are often examined in isolation. As a result, the ways in which they are organized into coherent mechanisms remain insufficiently understood. In particular, there is a lack of systematic frameworks that explain how multi-level factors interact and are translated into mathematical literacy outcomes through structured pathways.

### 2.3. Cross-Regional Perspectives: Mechanisms Underlying Differences Between East Asia and Western Contexts

From a comparative education perspective, differences in students’ mathematical learning motivation between East Asian and Western contexts are reflected not only in learning outcomes, but also in the structural organization of motivation and its culturally embedded functions. Overall, these two educational systems share certain cross-culturally stable mechanisms, while also exhibiting differentiated pathways shaped by cultural values, institutional arrangements, and classroom practices.

At the macro level, the positive role of intrinsic motivation in learning performance is consistently supported across regions, constituting a stable and universal mechanism. However, the functional role of extrinsic motivation varies significantly across cultural contexts. In East Asian educational cultures, norms of effort, responsibility, and achievement are often aligned with external evaluation systems, resulting in a cumulative or mutually reinforcing relationship between extrinsic and intrinsic motivation. In contrast, in Western systems that emphasize autonomy and interest-driven learning, excessive reliance on external rewards and sanctions may undermine intrinsic interest and autonomous engagement, and may even produce substitution effects (Zhu & Leung, 2011). Thus, the culturally contingent role of extrinsic motivation represents a key mechanism that differentiates these educational systems.

At the meso level, educational institutions and classroom practices further reinforce these differentiated pathways. East Asian classrooms tend to emphasize foundational training and the completion of structured tasks, whereas Western classrooms place greater emphasis on inquiry-based learning and open-ended problem solving. These pedagogical orientations are closely coupled with motivational structures, shaping students’ learning strategies and developmental trajectories (Zhao & Glewwe, 2010). In addition, teachers’ professional cultures and instructional beliefs play an important mediating role in the formation of motivation. Cross-cultural differences in teachers’ knowledge systems and pedagogical beliefs reflect deeper interactions among motivation, instruction, and cultural context (Kaiser & Blömeke, 2013).

At the micro level, individual differences in ability self-concept, task value, and developmental trajectories reflect the socio-cultural embeddedness of motivation. Empirical evidence suggests that students in East Asian and Western contexts exhibit distinct age- and gender-related patterns in motivational development, which are closely associated with processes of socialization and sustained exposure to different learning environments (Wang et al., 2020).

Taken together, while the commonality between East Asian and Western students lies in the stable positive role of intrinsic motivation, the differences primarily reside in the functional positioning of extrinsic motivation and its interaction with intrinsic motivation. Situating this “commonality–difference” structure within a multi-level explanatory framework, spanning macro-level cultural values, meso-level institutional and instructional practices, and micro-level psychological processes, reveals that learning motivation is not a singular psychological attribute, but rather the product of interactions among culture, institutions, and pedagogical practices.

### 2.4. Methodological Perspectives: Traditional and Emerging Approaches

Educational research has traditionally relied on quantitative paradigms, employing statistical modeling approaches such as regression analysis, hierarchical linear modeling (HLM), and structural equation modeling (SEM) to examine the relationships among background characteristics, psychological factors, and learning outcomes (Bryk & Raudenbush, 1992; Hanushek, 1979; Kaplan, 2009). These approaches have provided important insights into variable-level effects and latent structures, contributing to a well-established methodological foundation.

However, traditional statistical methods are often constrained by assumptions of linearity, additivity, and model specification. While effective for testing predefined theoretical relationships, they are limited in their ability to capture complex nonlinear interactions and high-dimensional dependencies among variables. Consequently, such approaches tend to emphasize isolated effects rather than the structural organization of learning mechanisms across multiple levels.

In recent years, machine learning approaches have been increasingly adopted in educational research, particularly in the analysis of large-scale assessment data. Compared with traditional models, machine learning techniques offer greater flexibility in modeling nonlinear relationships and detecting complex interaction patterns. Ensemble methods such as random forests and gradient boosting have demonstrated strong predictive performance and robustness in educational data contexts (Breiman, 2001).

At the same time, advances in interpretability frameworks, such as SHAP, have begun to address the limitations of “black box” models by enabling the decomposition of model predictions into the contributions of individual variables (Lundberg & Lee, 2017). These developments create new opportunities to move beyond prediction and toward the analysis of how multiple factors jointly contribute to learning outcomes.

Nevertheless, a key challenge remains: many machine learning approaches, despite their predictive power, still provide limited insight into the underlying mechanisms that generate observed outcomes. This tension between predictive performance and theoretical interpretability highlights the need for methodological approaches that can integrate both dimensions.

To address this gap, this study adopts an interpretable machine learning framework, aiming not only to identify key predictors of mathematical literacy but also to reveal how these factors are organized into structured learning mechanisms across different educational contexts.

## 3. Integrated Mechanism Framework

### 3.1. Conceptualizing Mechanisms in Mathematics Achievement

In the present study, the term “learning intelligence” does not refer to artificial intelligence in the computational sense. Rather, it refers to the organizational structure through which human learning processes, psychological regulation, contextual conditions, and behavioral enactment become coordinated within educational systems. Artificial intelligence methods are employed analytically to characterize these organizational patterns; however, the underlying structures examined in this study remain educational and psychological in nature rather than algorithmic in form.

Likewise, the term “mechanism” is not used to denote a verified causal process in the strict experimental or intervention-based sense. Instead, it refers to a structured organizational pattern through which contextual, psychological, and behavioral factors become systematically interconnected in shaping mathematics achievement. Accordingly, mechanisms are interpreted analytically rather than causally.

This distinction is particularly important because predictive importance, latent pathways, and interpretable machine learning patterns should not be conflated with causal explanation. SHAP values characterize the relative contribution and structural concentration of predictors within predictive models, whereas Structural Equation Modeling (SEM) captures theoretically informed relational pathways among latent constructs. Neither approach alone establishes causal mechanisms. Rather, the present study adopts a mechanism-oriented analytical perspective that integrates predictors, latent structures, and interpretable model patterns to examine how learning systems are organized across educational contexts.

From this perspective, the contribution of this study lies not merely in identifying influential predictors of mathematical literacy, but in examining how these predictors are hierarchically organized and structurally coordinated within broader learning systems. The study therefore emphasizes the organizational architecture of learning processes rather than isolated variable effects alone.

### 3.2. Cognitive Learning Systems as Organizational Structures

In the present study, Cognitive Learning Systems (CLS) are conceptualized as organized configurations through which contextual conditions, psychological regulation, learning behaviors, and educational environments become coordinated in shaping mathematical literacy. Rather than referring to artificial intelligence systems or computational architectures, CLS describes the organizational structure of human learning processes as they operate within broader educational systems.

From this perspective, mathematical achievement is not viewed as the product of isolated variables or independent psychological traits alone. Instead, it emerges through the interaction and coordination of multiple learning components, including socio-cultural conditions, motivational organization, self-regulation, instructional environments, and behavioral enactment.

Accordingly, the concept of Cognitive Learning Systems provides the theoretical foundation for integrating contextual mechanisms, psychological organization, and learning-process pathways within a unified analytical framework. Artificial intelligence methods are employed analytically to characterize these organizational structures, whereas the systems themselves remain educational and psychological in nature.

### 3.3. Theoretical Integration of EVT, SDT, and SRL

To explain how mathematical literacy emerges through coordinated learning processes, this study integrates Expectancy–Value Theory (EVT), Self-Determination Theory (SDT), and Self-Regulated Learning Theory (SRL) into a unified analytical framework.

EVT explains how students develop achievement expectations and subjective task values, thereby shaping their willingness to engage in learning activities (Eccles & Wigfield, 2002). Self-Determination Theory complements this perspective by explaining how motivational orientations become internalized and regulated through varying degrees of autonomy, competence, and psychological need satisfaction.

While EVT primarily addresses the formation of expectancy and value, SDT emphasizes the quality and regulation of motivation underlying sustained engagement.

Building upon these motivational foundations, SRL explains how psychological states are translated into observable learning behaviors, including strategic learning, metacognitive regulation, persistence, and engagement (Zimmerman, 2000).

In this sense, SRL represents the behavioral enactment layer within the broader learning process.

Accordingly, the three theories are not treated as parallel or additive explanations. Rather, they are conceptualized as sequentially connected and functionally complementary layers within a mechanism-oriented framework. EVT and SDT provide the motivational and psychological foundations for learning, whereas SRL explains how these internal states are operationalized into learning processes and academic behaviors.

Although these theories originate from different traditions within educational psychology, they collectively suggest that academic achievement is shaped through interconnected processes involving expectations, motivational regulation, self-beliefs, and strategic learning behaviors.

This integrative perspective provides a theoretical basis for examining how contextual conditions are translated into academic outcomes through multi-level psychological and behavioral organization.

### 3.4. A Multi-Layer Framework of Mathematical Literacy Formation

**Figure 1:**
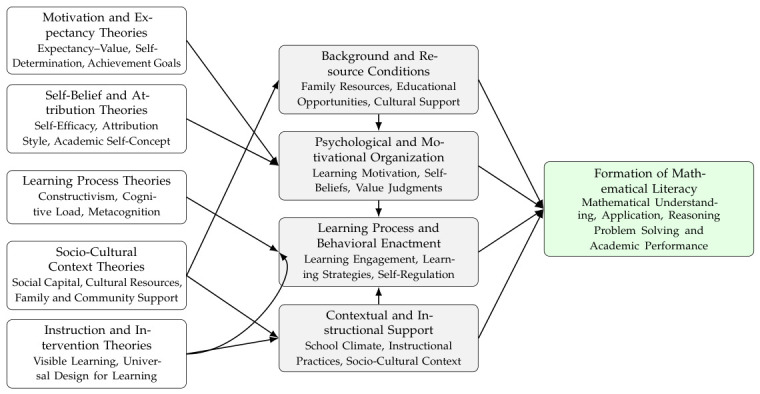
An Integrated Multi-Layer Framework of Mathematical Literacy Formation.

Building upon the mechanism-oriented perspective outlined above, Figure 1 presents an integrated theoretical framework for understanding how mathematical literacy is formed.

Rather than treating academic achievement as the result of isolated predictors, the framework conceptualizes mathematical literacy as an emergent outcome shaped through the interaction of contextual conditions, psychological processes, learning behaviors, and instructional environments.

To capture these multiple layers of influence, the framework synthesizes theories of motivation and expectancy (Eccles & Wigfield, 2002), self-beliefs and attribution (Bandura, 1997), learning processes (Zimmerman, 2000), socio-cultural contexts (Bourdieu, 1986), and instructional practices (Hattie, 2009).

These perspectives are incorporated not as independent explanatory models, but as interconnected components that contribute to different levels of the learning system.

Within this framework, each theoretical perspective contributes to a different layer of the learning system. Motivation and expectancy theories primarily explain how students develop academic goals, expectations, and value orientations that influence engagement (Eccles & Wigfield, 2002). Self-belief and attribution theories further explain how students interpret their academic competence and learning experiences, thereby shaping persistence, confidence, and achievement-related behaviors (Bandura, 1997).

Learning process theories focus on the behavioral and cognitive enactment of learning, including strategic learning, metacognitive regulation, and self-regulated engagement (Zimmerman, 2000). Socio-cultural theories emphasize that these individual processes are embedded within broader structural conditions, such as family resources, cultural capital, and institutional opportunity structures (Bourdieu, 1986). Instructional theories also highlight the role of teaching quality, learning environments, and feedback systems in facilitating or constraining learning development (Hattie, 2009).

Taken together, these perspectives suggest that mathematical literacy emerges through the interaction of four interrelated mechanism-oriented layers:

1. background and resource conditions,
2. psychological and motivational organization,
3. learning-process enactment, and
4. contextual and instructional support.

These layers operate dynamically rather than independently, collectively shaping students’ mathematical reasoning, problem solving, and academic performance.

### 3.5. Mechanism-Oriented Learning Pathways Across Contexts

**Figure 2:**
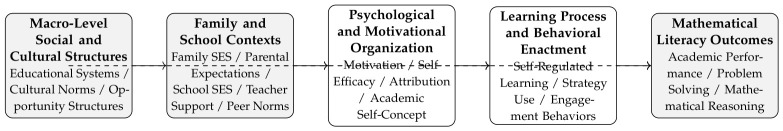
A Mechanism-Oriented Multi-Level Pathway Model of Mathematical Literacy.

Building upon the integrated framework presented above, Figure 2 presents a pathway-oriented representation of the proposed analytical structure.

The model conceptualizes the formation of mathematical literacy as a multi-level organizational process that progresses from macro-level socio-cultural structures to family and school contexts, then to psychological organization and learning processes, and ultimately to academic outcomes.

The model emphasizes that contextual conditions do not mechanically determine achievement outcomes. Rather, their effects are primarily mediated through psychological organization and behavioral enactment processes.

In this sense, learning systems are viewed as structured pathways through which resources, expectations, motivations, and learning behaviors become coordinated within broader educational environments.

Importantly, these pathways should not be interpreted as universally fixed or culturally deterministic structures.

Instead, they represent analytically inferred organizational tendencies observed within particular educational systems under the present modeling framework. Different high-performing systems may therefore achieve similar academic outcomes through structurally distinct learning organizations.

The pathway model further suggests that high-performing educational systems may differ not only in the magnitude of influential predictors, but also in the structural organization of learning pathways.

Some systems may exhibit relatively convergent structures in which psychological mediation plays a highly centralized role, whereas others may display more distributed or parallel organizational patterns that involve multiple learning processes operating simultaneously.

These distinctions should not be interpreted as fixed cultural properties or as universal characteristics of East Asian or Western societies.

Rather, they represent analytical tendencies inferred from the present comparative framework and may vary across institutional and educational contexts.

### 3.6. Research Gaps and Analytical Motivation

Despite substantial research on mathematics achievement and educational inequality, several important limitations remain in the existing literature.

First, prior studies often focus on isolated levels of analysis, such as socio-economic status, motivation, or learning strategies, without sufficiently examining how these factors become structurally interconnected within broader learning systems.

As a result, the organizational pathways linking contextual conditions, psychological organization, and learning behaviors remain insufficiently understood.

Second, although large-scale assessments such as PISA have documented substantial cross-regional differences in mathematics performance, relatively limited attention has been paid to whether high-performing systems achieve similar outcomes through structurally distinct learning organizations.

In particular, systematic evidence regarding variations in pathway concentration, psychological mediation, and multi-level coordination across educational contexts remains limited.

Third, although existing educational and psychological theories have proposed various learning processes and organizational patterns associated with academic achievement, their applicability across different educational systems has rarely been examined within an integrated comparative framework that combines interpretable machine learning and structural pathway analysis.

### 3.7. Research Questions and Analytical Expectations

To address these gaps, the present study develops a mechanism-oriented analytical framework that integrates contextual conditions, psychological organization, learning processes, and academic outcomes.

Using PISA 2018 data, the study conducts cross-regional comparisons among high-performing educational systems to examine how different forms of learning-system organization are associated with mathematics achievement.

Rather than testing strict causal hypotheses, the study is guided by the following research questions and analytical expectations:

1. Across different educational systems, which background, attitudinal, and learning-related factors play a key role in shaping students’ mathematical literacy?
2. To what extent do the importance and effects of these factors differ across high-performing countries and regions, and what structural patterns characterize these differences?
3. What are the key components and organizational structures of the mechanisms underlying mathematical literacy across different educational systems?

The study expects that high-performing educational systems may achieve comparable levels of mathematics performance through structurally different organizational pathways rather than through identical configurations of influential predictors.

By integrating interpretable machine learning with theory-informed structural analysis, the framework aims to provide a more systematic understanding of how learning systems organize the translation of contextual resources into academic outcomes.

## 4. Data and Methodology

### 4.1. Data Source and System Selection

This study utilizes student-level data from the Programme for International Student Assessment (PISA) 2018 released by the OECD. PISA is one of the most widely used international large-scale assessment programs and provides extensive information on students’ academic achievement, socio-economic background, learning environments, psychological characteristics, and learning behaviors. The dataset offers strong cross-national comparability and rich contextual information, thereby providing an important empirical foundation for examining learning-system organization across educational contexts.

The present study focuses specifically on high-performing educational systems. The objective is not to compare successful and unsuccessful systems within a deficit-oriented framework, but rather to investigate whether comparable levels of mathematical achievement may emerge through structurally distinct learning organizations. By restricting the analysis to relatively high-performing systems, the study seeks to reduce extreme achievement disparities while highlighting differences in pathway organization, psychological mediation, and learning-process coordination across contexts.

Educational systems were selected based on three criteria: (1) high performance in the OECD PISA 2018 mathematics assessment, (2) sufficient sample size and questionnaire completeness for comparative modeling, and (3) the availability of key contextual, psychological, and behavioral variables required for the proposed analytical framework. Although several additional high-performing systems are presented in the descriptive overview, only regions with sufficiently complete variable structures and stable estimation conditions were retained for subsequent comparative analyses.

For descriptive purposes, Table 1 summarizes the sample size, number of retained variables, and average mathematics performance for each educational system.

In terms of sample structure, most East Asian and European systems contain approximately 4700 to 6700 students, whereas Canada includes a substantially larger sample exceeding 22,000 students. This variation provides different levels of statistical stability for predictive modeling and robustness evaluation.

There is also substantial variation in variable coverage across educational systems. For example, Korea and Estonia contain particularly rich questionnaire structures, whereas other regions include more limited contextual information. Such differences may influence both model complexity and the interpretability of pathway structures across regions.

In terms of mathematics performance, the selected systems largely correspond to the upper range of the OECD PISA 2018 rankings. East Asian systems dominate the highest positions, with China (B–S–J–Z) and Singapore forming the top-performing group, followed by Macao, Hong Kong, Chinese Taipei, Japan, and Korea. Among Western systems, Estonia, the Netherlands, Poland, and Canada also demonstrate relatively high levels of achievement.

This cross-regional configuration provides an important analytical opportunity. Rather than assuming that high academic performance emerges through identical educational processes, the present study investigates whether different educational systems may achieve similar outcomes through structurally distinct learning organizations and pathway configurations.

### 4.2. Sample Composition and Descriptive Characteristics

Building upon the data overview above, this section further describes the composition and descriptive characteristics of the final analytical sample. The objective is to establish the empirical context necessary for subsequent predictive modeling and structural pathway analysis.

Following preprocessing and variable-selection procedures, the final analytical sample included multiple high-performing educational systems across East Asia, Europe, and North America. Sample size varies substantially across regions, reflecting differences in PISA sampling procedures, participation structures, and questionnaire completeness. Such variation may also influence model stability, predictive differentiation, and pathway estimation across educational contexts.

Following the integrated mechanism-oriented framework proposed in the previous section, variables were organized into four broad analytical layers: (1) contextual and socio-economic conditions, (2) psychological and motivational organization, (3) learning-process and behavioral enactment, and (4) academic outcomes.

Table 2 summarizes the major dimensions and representative constructs derived from the PISA student questionnaire framework.

Although all selected systems belong to the upper range of global PISA mathematics performance, meaningful variation remains across regions. East Asian systems tend to exhibit relatively higher mean achievement and more concentrated score distributions, whereas high-performing Western systems show slightly lower mean scores alongside greater internal heterogeneity. These patterns suggest that even among successful educational systems, the structural organization of achievement may differ substantially.

Regional differences are also observed in socio-economic conditions, learning motivation, self-beliefs, learning behaviors, and school climate variables. While such differences should not be interpreted as direct causal explanations, they provide an important descriptive foundation for understanding variations in predictor concentration, pathway organization, and structural mediation across educational systems.

### 4.3. Treatment of PISA Design Features

As an international large-scale assessment, PISA adopts a complex sampling design involving stratified sampling procedures, student weights, and plausible values. In the present study, the analytical framework primarily emphasizes comparative predictive modeling and mechanism-oriented structural interpretation rather than strict population-parameter estimation following the OECD reporting tradition.

To improve cross-regional comparability, sampling weights and mathematics plausible values were incorporated during preprocessing and comparative analysis stages where appropriate. However, replicate-weight variance estimation procedures were not fully implemented throughout all machine learning stages because the primary objective of the study was predictive differentiation and structural interpretation rather than precise population-level inference.

Accordingly, the findings should be interpreted primarily as comparative analytical evidence regarding learning-system organization rather than exact population estimates of educational effects. This distinction is particularly important because the study focuses on identifying structurally distinct learning pathways across educational systems rather than producing official OECD-style population statistics.

### 4.4. Missing Data Treatment and Preprocessing

Before model estimation, missing-data patterns were systematically examined across regions and variable categories. Preliminary inspection suggested that missingness was primarily associated with questionnaire-response patterns and variable-specific nonresponse rather than with systematic exclusion based solely on mathematics performance. Variables with extremely high missingness or insufficient cross-regional comparability were removed prior to modeling to improve stability and interpretability.

Imputation procedures were conducted separately within each educational system to preserve region-specific distributional characteristics and avoid distortions arising from pooled imputation across heterogeneous contexts. This region-specific approach was intended to maintain the distributional features of each educational system while ensuring comparability in subsequent modeling.

The objective of the imputation procedure was not to fully reproduce the uncertainty structure associated with multiple-imputation frameworks, but rather to provide a stable and comparable preprocessing strategy for predictive modeling and cross-regional structural analysis. The imputation procedure was primarily intended to support predictive comparability and stable structural interpretation across high-dimensional regional datasets rather than formal causal or population-level inference. Accordingly, the findings should be interpreted with appropriate caution regarding population-level inference.

After imputation, variables were standardized and screened for redundancy and multicollinearity. Principal Component Analysis (PCA) was subsequently applied to highly correlated variables to reduce dimensionality and improve cross-regional comparability. Beyond statistical reduction, PCA also facilitated the construction of theoretically meaningful latent dimensions, including learning motivation, psychological organization, learning behaviors, and contextual support structures.

### 4.5. Predictive Modeling and Interpretability Strategy

Building upon the mechanism-oriented comparative framework developed in the previous section, this study adopts a multi-stage analytical pipeline integrating machine learning, interpretable modeling, and structural pathway analysis.

A classification-oriented framework was adopted because the study focused on identifying structurally distinct proficiency-level organizations rather than predicting marginal score variation alone. This design aligns more directly with the comparative objective of examining how different educational systems organize learning processes associated with mathematics achievement.

Model evaluation was conducted using stratified train–test splitting combined with cross-validation procedures to improve robustness and reduce the risk of overfitting. Because proficiency categories may exhibit unequal distributions across educational systems, stratified sampling procedures were adopted during train–test splitting and cross-validation to reduce potential imbalance effects and maintain comparable category representation across modeling stages. Hyperparameter tuning was performed using grid-search strategies within the training data. Baseline benchmark models, including logistic regression and decision-tree classifiers, were additionally estimated to provide comparative reference points for evaluating predictive differentiation.

At the stage of variable identification and preliminary modeling, CatBoost was employed to analyze high-dimensional variables including contextual background, psychological characteristics, and learning behaviors. By leveraging gradient boosting and ordered encoding strategies, CatBoost characterizes influential predictor structures associated with mathematics achievement while improving robustness in heterogeneous high-dimensional environments.

To enhance interpretability and capture nonlinear relationships, Random Forest combined with SHAP (Shapley Additive Explanations) was subsequently employed. SHAP analysis was used to characterize relative contribution patterns, predictor concentration, and structural dispersion within predictive models rather than to establish causal mechanisms. This approach allows for both global and local interpretation of predictor organization across educational systems.

**Figure 3:**
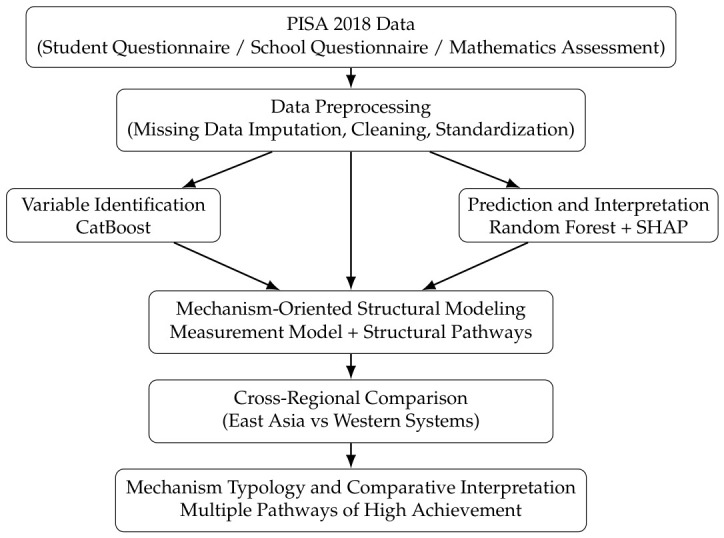
Analytical Pipeline and Mechanism-Oriented Comparative Framework of the Study.

Figure 3 visualizes the analytical pipeline adopted in the study. Data preprocessing forms the methodological foundation, followed by two complementary analytical branches: variable identification through CatBoost and interpretable predictive analysis through Random Forest and SHAP. These components jointly inform the subsequent structural modeling stage.

Overall, the methodological pathway can be summarized as follows: data preprocessing → variable organization → predictive modeling → interpretable analysis → structural pathway modeling → cross-regional comparison. Machine learning and SEM approaches play complementary roles within this framework. Whereas machine learning emphasizes predictive differentiation and structural characterization, SEM provides theory-informed pathway organization and comparative structural interpretation.

Importantly, predictive differentiation and explanatory interpretation serve distinct purposes within the present framework. Machine learning models were used to characterize predictive organization and structural concentration, whereas SEM was employed to examine theory-informed pathway relationships. Neither predictive importance nor SHAP contribution values should be interpreted as direct evidence of causal necessity.

### 4.6. AI-Assisted Variable Organization and Structural Specification

The transition from interpretable machine learning analysis to SEM specification was guided by a theory-informed rather than purely data-driven strategy. Variables were not transferred directly from predictive models into SEM solely on the basis of SHAP importance rankings or predictive contribution scores. Instead, machine learning results were used primarily to characterize comparative predictor organization, identify structurally influential variable groups, and support the refinement of theoretically meaningful latent dimensions.

The SEM structures remained theoretically grounded throughout the modeling process. Latent dimensions and pathway structures were initially derived from the integrated mechanism-oriented framework developed in the previous section, including contextual conditions, psychological organization, learning-process enactment, and academic outcomes. Machine learning analyses subsequently informed the prioritization, refinement, and comparative interpretation of variables within these broader theoretical categories.

Accordingly, SHAP analysis was used primarily as an interpretability and organizational-support tool rather than as an independent mechanism-discovery procedure. The objective was not to allow machine learning algorithms to generate latent structures autonomously, but rather to integrate predictive differentiation with theory-informed structural interpretation.

At the same time, the study acknowledges that alternative feature-selection pipelines or different preprocessing strategies could potentially yield partially different organizational patterns. Therefore, the resulting pathway structures should be interpreted as analytically inferred and comparatively informative rather than uniquely determined representations of educational systems.

### 4.7. Structural Equation Modeling Specification

Based on the latent structures identified through PCA and theory-informed variable organization, Structural Equation Modeling (SEM) was employed to estimate the multi-level relationships among contextual conditions, psychological organization, learning behaviors, and academic outcomes.

The SEM framework combined measurement models and structural pathway models. Measurement models were first evaluated to assess construct validity, factor-loading adequacy, and convergence properties. Models were estimated using robust maximum likelihood estimation to improve stability under non-normality and heterogeneous variable distributions commonly observed in large-scale educational survey data.

Model evaluation considered multiple fit indices, including the Comparative Fit Index (CFI), Tucker–Lewis Index (TLI), Root Mean Square Error of Approximation (RMSEA), and Standardized Root Mean Square Residual (SRMR). Following commonly used guidelines in SEM research (Hooper et al., 2008), CFI and TLI values approaching 0.90 and RMSEA and SRMR values below approximately 0.08 were interpreted as indicating acceptable model fit. Because fit indices are sensitive to model complexity, sample size, and cross-regional heterogeneity in large-scale educational datasets, model adequacy was evaluated comprehensively rather than relying on rigid cutoff thresholds alone.

Structural models were subsequently estimated to examine the organization of pathway relationships across mechanism layers.

The proposed SEM structure follows the pathway sequence developed in the integrated framework section: contextual conditions → psychological organization → learning-process enactment → mathematics achievement. Both direct and indirect relationships among mechanism layers were estimated to capture the coordinated organization of learning systems.

Because the primary objective of the study was comparative structural interpretation rather than strict latent mean equivalence testing, full measurement invariance across all educational systems was not rigidly imposed. Accordingly, the SEM results should be interpreted primarily as comparative analytical evidence regarding pathway organization rather than exact population-equivalent structural estimates.

Overall, SEM serves as the central mechanism-oriented modeling component within the analytical framework, linking theory-informed latent structures with cross-regional pathway interpretation.

## 5. Results

### 5.1. Predictive Performance and Comparative Classification Patterns

This section reports the overall classification performance of the CatBoost model across educational systems, with the objective of evaluating its ability to differentiate mathematics proficiency categories across regions. Students’ mathematics scores were categorized into multiple performance groups, and a supervised classification framework was employed to predict category membership. Model performance was evaluated using Accuracy, Macro-average F1, and Weighted-average F1 to account for differences in class distribution and potential imbalance across proficiency categories.

To provide a comparative benchmark for evaluating predictive differentiation, CatBoost performance was compared with baseline classification approaches, including Logistic Regression and Random Forest classifiers. The benchmark models were estimated using the Poland sample as a representative high-performing educational system under the same classification framework.

The benchmark comparison across classification models for the Poland sample is reported in Table 3. Compared with baseline classification models, CatBoost demonstrated comparatively stronger predictive differentiation across proficiency categories under heterogeneous educational conditions. The improvement over simpler models suggests that nonlinear interactions and complex feature organization contribute meaningfully to the classification structure observed across educational systems.

Table 4 summarizes the classification performance of the CatBoost model across educational systems.

Overall, the model demonstrates moderate-to-strong predictive differentiation across educational systems, with classification accuracy ranging from approximately 0.69 to 0.87. The relative consistency between Accuracy and Weighted F1 suggests comparatively stable classification performance despite variation in sample size and class distribution across regions. By contrast, lower Macro F1 values observed in several systems indicate greater difficulty in distinguishing certain proficiency groups under more heterogeneous classification conditions.

From a cross-regional perspective, predictive differentiation appears comparatively stronger in educational systems such as Korea, Poland, and Canada, where both Accuracy and Weighted F1 exceed 0.85. This pattern may indicate that the predictor organization associated with mathematics proficiency is comparatively more differentiated within these educational systems. In contrast, regions such as Shanghai and Hong Kong exhibit lower Macro F1 values, suggesting greater overlap among some proficiency categories, particularly among lower-performing students.

At the category level, the model generally performs best in identifying medium-performing students, followed by high-performing students, whereas classification performance for low-performing groups tends to be comparatively weaker in several educational systems. This pattern likely reflects both smaller subgroup sample sizes and greater heterogeneity among lower-performing students. In addition, variability in F1 scores for high-performing groups across educational systems may further suggest that the structural organization associated with high achievement differs across contexts.

Notably, these findings should not be interpreted as evidence of causal mechanisms. Rather, the results characterize comparative patterns of predictive differentiation across educational systems. The observed variation in classification performance may nevertheless suggest that the organizational structures associated with mathematics achievement differ across regions, including the extent to which contextual, motivational, and behavioral factors become structurally coordinated within learning systems.

This variability may further indicate that motivational and contextual factors are differently organized across educational systems, potentially challenging rigid distinctions between psychological and contextual dimensions in comparative educational research. In some systems, motivational organization may be more strongly embedded within broader family, institutional, or socio-cultural structures, whereas in others it may operate more independently at the individual level.

In the next section, SHAP-based interpretation and comparative feature-organization analysis will be employed to further examine how predictor concentration and structural organization vary across educational systems.

### 5.2. SHAP-Based Analysis of Predictor Organization and Structural Concentration

To comparatively characterize the organizational patterns associated with students’ mathematical achievement, this section applies interpretable machine learning methods based on Random Forest modeling and SHAP (Shapley Additive Explanations). Rather than treating feature importance as evidence of causal mechanisms, the analysis focuses on how predictor concentration and organizational structure differ across educational systems within the proposed analytical framework linking contextual conditions, psychological organization, learning processes, and academic outcomes.

Table 5 reports the comparative SHAP concentration ratios across educational systems. The SHAP results appear broadly consistent with the proposed comparative framework and provide additional evidence regarding how predictor concentration differs across educational systems. In several East Asian educational systems, predictive differentiation appears to rely more heavily on a comparatively limited subset of contextual and motivational predictors. These variables also exhibit relatively stable importance rankings across regions, suggesting comparatively concentrated organizational tendencies within the predictive structure.

By contrast, many Western educational systems display relatively more distributed predictor organization. Multiple predictor groups, including reading behaviors, learning processes, contextual variables, and psychological factors, simultaneously contribute to predictive differentiation across proficiency categories. In some educational systems, psychological and contextual variables also appear to become more structurally intertwined within the predictive organization, suggesting that the boundaries between motivational regulation and contextual influence may vary across learning environments.

To further characterize whether predictor organization exhibited concentrated or distributed tendencies, a SHAP concentration ratio was calculated based on the cumulative contribution of the top-ranked predictors within each educational system. Higher concentration ratios indicate that predictive differentiation relies more heavily on a limited subset of dominant predictors, whereas lower ratios suggest comparatively distributed predictor organization.

**Figure 4:**
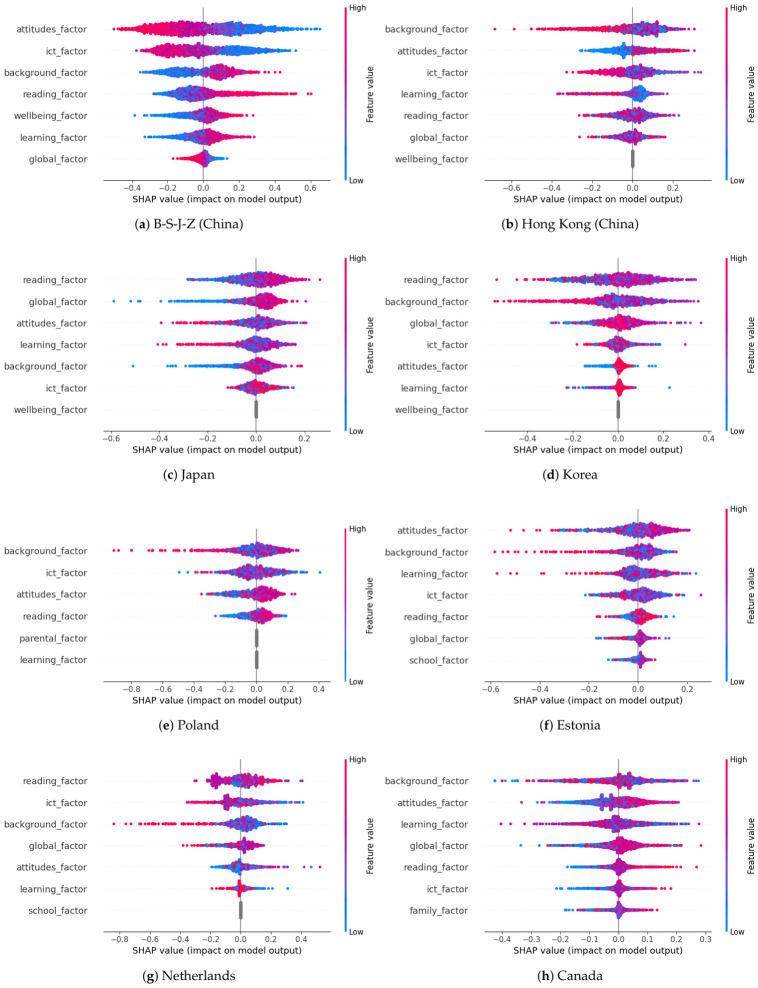
SHAP-based predictor-importance distributions across educational systems derived from Random Forest models.

Figure 4 presents the SHAP-based predictor-importance distributions across eight educational systems derived from Random Forest models. A visible contrast emerges between comparatively concentrated and distributed predictor structures. Several East Asian systems exhibit higher concentration ratios and more centralized predictor organization, whereas many Western educational systems display comparatively dispersed and heterogeneous importance distributions.

This distinction between comparatively concentrated and distributed predictor organization appears broadly consistent with the organizational tendencies identified in the proposed comparative framework. However, these findings should be interpreted analytically rather than causally. SHAP values indicate relative contributions within predictive models rather than causal necessity or universally invariant mechanisms.

Taken together, the results suggest that cross-regional variation in mathematical achievement may be associated less with the simple presence of specific predictors than with how contextual, psychological, and behavioral variables become comparatively coordinated within educational systems. Several East Asian educational systems appear to exhibit comparatively concentrated organizational tendencies, whereas many Western educational systems display relatively distributed and multi-path predictor organization.

### 5.3. Comparative Structural Pathway Patterns Across Educational Systems

To further examine cross-regional variation in pathway organization associated with mathematics achievement, this study applies a comparable structural equation modeling (SEM) framework across multiple high-performing educational systems and comparatively examines key standardized pathway coefficients as shown in Table 6.

Model fit varied across educational systems, with some regional models exhibiting comparatively weaker CFI or RMSEA values. These differences likely reflect cross-regional heterogeneity in questionnaire-response structures, institutional organization, and latent construct comparability. Accordingly, the pathway patterns reported here should be interpreted comparatively and analytically rather than as evidence of universally invariant latent architectures across contexts.

Overall, the results suggest substantial variation in pathway organization across educational systems. Rather than indicating fundamentally different sets of predictors, the findings primarily reflect differences in the relative coordination, concentration, and organization of contextual, psychological, and behavioral components within each educational system.

Several East Asian educational systems appear to exhibit comparatively more concentrated pathway organization. In Shanghai, educational expectations and family background display relatively strong standardized coefficients, suggesting comparatively stronger coordination between contextual expectations and motivational organization within the observed pathway structure. This pattern may indicate that achievement-related psychological regulation is more closely embedded within broader family and socio-cultural expectations.

Hong Kong exhibits a comparatively behavior-oriented organizational tendency, in which learning behaviors, learning time, and classroom practices demonstrate stronger direct associations with mathematics achievement. Japan displays a comparatively psychology-oriented pattern, with psychological composites showing relatively stronger pathway coefficients, suggesting a more centralized role for motivational and self-regulatory organization within the modeled structure.

Korea appears to exhibit a comparatively hybrid organizational tendency characterized by psychological coordination combined with behavioral reinforcement. Psychological variables retain relatively strong associations with achievement (β=0.167, p<0.001), while learning behaviors function as complementary organizational pathways. At the same time, time-investment variables display comparatively smaller coefficients, suggesting that pathway coordination may depend more heavily on motivational organization and behavioral efficiency than on time allocation alone.

In several educational systems, motivational variables also appear to be closely embedded within contextual and family-related structures rather than operating as entirely independent psychological dimensions. This pattern may help explain why the distinction between contextual and psychological organization varies across educational contexts.

By contrast, several European educational systems appear to exhibit comparatively more distributed pathway organization. Estonia demonstrates comparatively lower pathway concentration, with fewer highly centralized pathways observed within the model structure. Poland likewise displays comparatively distributed organizational tendencies, in which multiple predictor groups contribute without forming a single dominant pathway configuration.

Norway and Canada exhibit comparatively behaviorally mediated organizational tendencies, in which learning behaviors function as important transmission channels within the pathway structure. However, the sources associated with these pathways differ across systems. In Norway, digital behavior appears to become associated with achievement primarily through reading-related pathways, whereas in Canada, reading behaviors appear more closely coordinated with broader financial and contextual learning structures. In addition, several digital pathways exhibit unstable or partially negative associations, suggesting that digital engagement may operate differently across educational environments.

Taken together, the comparative SEM results suggest that high-performing educational systems may exhibit partially distinct forms of pathway organization associated with mathematics achievement. Several systems appear comparatively concentrated around a limited number of strongly coordinated pathways, whereas others exhibit relatively distributed and multi-path organizational tendencies.

These comparative pathway patterns also appear broadly consistent with the principle of equifinality, suggesting that similar levels of high performance may emerge through partially different forms of contextual, psychological, and behavioral coordination. Accordingly, cross-regional variation in mathematics achievement may be associated less with the mere presence of particular predictors than with how these factors become comparatively organized within educational systems.

## 6. Discussion

### 6.1. From Predictors to Organizational Learning Structures

A central contribution of this study lies in shifting the analytical focus from identifying influential predictors to comparatively characterizing the structural organization associated with learning processes across educational systems. Prior research on mathematical achievement has often emphasized the relative importance of individual variables, such as socioeconomic status, motivation, or learning strategies. However, the present findings suggest that predictor importance alone may be insufficient for understanding how achievement-related differences become comparatively organized within broader learning systems.

By integrating machine learning models, SHAP-based interpretation, and structural equation modeling, the study demonstrates that contextual, psychological, and behavioral dimensions appear interconnected within broader organizational structures rather than functioning as isolated influences. Predictors identified through CatBoost and Random Forest modeling do not simply operate independently; instead, they appear embedded within comparatively coordinated pathway structures linking contextual conditions, psychological regulation, learning behaviors, and academic outcomes.

The SHAP analysis further suggests that similar predictors may exhibit substantially different importance distributions across educational systems. This indicates that the same variables may play partially different organizational roles depending on how they are embedded within broader educational and socio-cultural contexts. SEM results are broadly consistent with this interpretation by showing that many contextual and psychological dimensions are associated with achievement through indirect and mediated pathway relationships, particularly through learning-related behavioral organization.

Taken together, these findings support a more organization-oriented interpretation of mathematical literacy. From this perspective, predictive modeling serves not as evidence of fixed causal mechanisms, but as a tool for comparatively characterizing how learning-related dimensions become coordinated within educational systems. Importantly, the present study does not interpret predictive importance or SEM pathways as evidence of universally invariant causal mechanisms. Rather, the findings are interpreted as comparatively inferred organizational patterns through which contextual, psychological, and behavioral dimensions become associated within high-performing educational systems.

### 6.2. The Organizational Role of Self-Regulated Learning

Across both machine learning and structural modeling results, self-regulated learning appears consistently associated with pathway coordination across educational systems. Rather than functioning only as an isolated predictor, self-regulated learning appears closely associated with the integration of contextual conditions, motivational regulation, and learning engagement within broader learning structures.

In the SHAP analysis, variables related to reading behaviors, learning strategies, sustained engagement, and behavioral regulation consistently appear among the more important predictors across educational systems. These variables frequently occupy central positions within the predictive structure, suggesting that they may play an integrative role in linking contextual inputs and psychological dimensions.

SEM results further support this interpretation by showing that many contextual and motivational variables are associated with achievement indirectly through learning-related behaviors. In several educational systems, the relationship between family background, educational expectations, and achievement appears coordinated through behavioral engagement, particularly reading-related activities and self-regulated learning strategies. This pattern suggests that self-regulated learning may function as an important organizational component within the broader learning architecture.

At the same time, the organizational positioning of self-regulated learning appears to differ across educational systems. In several East Asian contexts, self-regulated learning appears embedded within comparatively sequential and concentrated pathway structures associated with normative expectations and disciplined learning organization. By contrast, in many Western systems, self-regulated learning appears more flexibly coordinated with multiple parallel contextual and psychological inputs.

These findings may therefore support a broader interpretation of self-regulated learning as an organizational component linking contextual, motivational, and behavioral dimensions within educational systems. Such an interpretation extends beyond viewing self-regulation merely as an individual competency and instead situates it within the broader structural coordination of learning processes.

### 6.3. Cross-Regional Variability in Learning-System Organization

The findings of this study suggest that high-performing educational systems may exhibit partially distinct forms of organizational coordination associated with mathematical literacy. Traditional discussions of high achievement often emphasize common explanatory factors, such as strong family support, high expectations, or effective instructional practices. However, the present findings indicate that similar levels of achievement may emerge through different forms of contextual, psychological, and behavioral coordination.

Several East Asian educational systems appear comparatively more concentrated in predictor and pathway organization. In these systems, contextual resources, educational expectations, and behavioral regulation appear more tightly coordinated within comparatively sequential pathway structures. By contrast, many Western educational systems and Canada appear comparatively more distributed in both predictor concentration and pathway organization, with multiple contextual, instructional, cognitive, and psychological dimensions contributing simultaneously to achievement-related differentiation.

Importantly, these distinctions should not be interpreted as rigid cultural classifications or deterministic educational models. Considerable variation remains within both East Asian and Western systems, and the observed patterns represent comparative analytical tendencies rather than universally fixed structures. Nevertheless, the results appear broadly consistent with the principle of equifinality, suggesting that similar educational outcomes may emerge through different forms of organizational coordination.

One particularly notable finding concerns the relationship between contextual and psychological dimensions. In several educational systems, motivational variables appear closely embedded within family-related and contextual structures rather than functioning as entirely independent psychological constructs. This pattern may suggest that the organizational boundaries between contextual influence and psychological regulation are not equally differentiated across educational systems. From this perspective, interpretable machine learning may contribute not only to predictive differentiation, but also to a reconsideration of conventional disciplinary distinctions between contextual and psychological organization.

More broadly, the findings imply that cross-national differences in achievement may be associated less with the simple presence of particular variables than with how these variables become structurally coordinated within educational systems. Viewing high-performing systems through this organizational perspective provides a more nuanced interpretation of educational effectiveness and highlights the importance of context-sensitive analytical frameworks in comparative educational research.

### 6.4. Connections to Existing Literature

The findings of this study contribute to a growing body of research examining mathematical literacy as a multidimensional and contextually embedded process rather than as the product of isolated variables. Consistent with prior literature, the results support the importance of self-regulated learning, motivational organization, family background, instructional conditions, and socio-cultural context in shaping mathematics-related outcomes.

Previous studies have shown that self-regulated learning plays an important role in sustaining effort and supporting mathematical reasoning (Gabriel et al., 2020). The present findings extend this literature by suggesting that self-regulated learning may also occupy a comparatively central organizational position within broader pathway structures, particularly in several high-performing East Asian systems where contextual expectations, motivation, and learning behaviors appear more tightly coordinated.

At the family level, earlier research has documented differentiated effects of home learning environments and parental involvement (Skwarchuk et al., 2022). The current findings are broadly consistent with this perspective by suggesting that contextual resources operate through selective and comparatively mediated organizational patterns rather than through uniform direct effects.

Similarly, prior research on instructional practices has emphasized the role of cognitive activation, classroom engagement, and meaningful mathematical tasks in supporting mathematical literacy (H. Zhang, 2018). The present study further suggests that these instructional dimensions may not function independently, but instead become integrated within broader organizational structures involving contextual support, psychological regulation, and behavioral coordination.

From a socio-cultural perspective, existing literature has emphasized that mathematical literacy is embedded within broader cultural and social contexts (Croce, 2020). The present findings support this interpretation by suggesting that socio-cultural organization may shape how contextual, motivational, and behavioral dimensions become coordinated across educational systems.

Methodologically, the study also contributes to emerging work applying interpretable machine learning within educational research. While machine learning approaches have increasingly been used for predictive differentiation (Gao & Li, 2024), their potential for comparatively characterizing organizational patterns has remained relatively underexplored. By integrating SHAP-based interpretation with structural equation modeling, the present study demonstrates how machine learning methods can complement theory-informed pathway analysis within comparative educational research.

Overall, the findings suggest that understanding mathematical literacy may require moving beyond variable-centered explanations toward a broader interpretation of learning as a comparatively coordinated and multi-level organizational process.

## 7. Conclusions

### 7.1. Key Findings

This study examined the organizational patterns associated with mathematical literacy across high-performing educational systems by integrating interpretable machine learning and structural equation modeling. Rather than treating achievement as the result of isolated predictors, the study adopted a mechanism-oriented analytical perspective to examine how contextual, psychological, and behavioral dimensions become comparatively coordinated within educational systems.

Three key findings emerged. First, academic achievement-related differences are not fully explained by the presence of individual predictors alone, but are associated with how predictors are organized into broader structural patterns. Although similar categories of variables appear across educational systems, their relative importance, pathway positions, and coordination patterns differ substantially.

Second, self-regulated learning appears to play an important organizational role in linking contextual conditions, psychological regulation, and academic outcomes. Rather than functioning only as an independent predictor, self-regulated learning is closely associated with the coordination of background conditions, motivational factors, and learning behaviors within the observed pathway structures.

Third, cross-regional comparisons suggest that high-performing educational systems may exhibit partially distinct organizational tendencies. Several East Asian systems appear comparatively more concentrated in pathway organization, whereas many Western systems and Canada display relatively more distributed and multi-path configurations. These patterns are broadly consistent with the principle of equifinality, suggesting that high performance may be associated with different forms of contextual, psychological, and behavioral coordination rather than a single universal model of achievement.

### 7.2. Implications

This study has several theoretical, methodological, and practical implications.

Theoretically, the findings contribute to the literature by shifting attention from the identification of influential predictors to the analysis of how learning-related factors are structurally organized. The results suggest that the same variables may play different roles depending on their position within a broader learning-system structure. This perspective provides a more nuanced understanding of cross-regional differences in mathematical literacy by emphasizing organizational patterns rather than isolated variable effects.

Methodologically, the study illustrates the value of combining interpretable machine learning with theory-informed structural modeling. Machine learning models, including CatBoost and Random Forest, are useful for characterizing high-dimensional predictor structures and identifying patterns of predictive differentiation. SHAP analysis further helps describe predictor concentration and distribution. However, these results should not be interpreted as direct evidence of causal mechanisms. SEM complements the machine learning analysis by providing a theory-informed representation of pathway relationships among latent dimensions. Together, these approaches support a comparative organizational interpretation of learning systems.

From a practical perspective, the findings suggest that educational policies and interventions should be context-sensitive rather than based on a single model of best practice. In comparatively concentrated systems, interventions targeting educational expectations, motivational regulation, and learning-behavior coordination may have broader relevance. In relatively distributed systems, multidimensional support involving reading engagement, digital literacy, classroom participation, and self-regulated learning may be more appropriate. Therefore, policy transfer across educational systems should consider not only whether a factor is important, but also how it is embedded within the broader learning structure.

### 7.3. Limitations and Future Research

Several limitations should be acknowledged.

First, this study is based on cross-sectional PISA 2018 data. Therefore, the analysis cannot establish temporal ordering or causal direction with certainty. The findings should be interpreted as associations and organizational patterns consistent with the proposed analytical framework, rather than as evidence of strict causal mechanisms. Future research using longitudinal data would be valuable for examining how these pathway structures develop over time.

Second, because the data were collected before major post-2020 transformations in educational technology and learning environments, the findings may not fully capture more recent changes in digital learning behavior, remote learning, or AI-supported educational systems. Future studies using more recent datasets could examine whether the organizational patterns identified here remain stable under newer educational conditions.

Third, although interpretable machine learning provides useful evidence regarding predictor concentration and relative contribution, SHAP values indicate contributions to prediction rather than causal necessity. Alternative feature-selection procedures, modeling specifications, or preprocessing strategies may produce partially different predictor structures. Future research could further test the robustness of these patterns across alternative machine learning pipelines.

Fourth, the SEM analysis depends on latent-variable construction and model specification decisions. Some regional models showed weaker fit indices than others, and full cross-group measurement invariance was not strictly established across all educational systems. Therefore, the SEM results should be interpreted comparatively and analytically rather than as evidence of universally invariant latent architectures. Future research could conduct more formal invariance testing and compare alternative structural specifications.

Fifth, this study focuses on high-performing educational systems. This design is appropriate for examining whether successful systems achieve similar outcomes through different organizational pathways, but it limits the generalizability of the findings to average-performing or low-performing contexts. Future studies could extend the framework to more socioeconomically diverse systems and educational contexts with different curricular, institutional, or policy structures.

Finally, while this study identifies comparative organizational patterns across regions, it does not fully capture the institutional, curricular, or classroom-level processes that may shape these patterns. Further research is needed to examine how policy design, curriculum structure, teacher practice, and student support systems interact with contextual, psychological, and behavioral dimensions at a more granular level.

Overall, this study provides a comparative organizational interpretation of mathematical literacy across high-performing educational systems. By integrating data-driven modeling with theory-informed structural analysis, it offers a framework for understanding how contextual, psychological, and behavioral factors may become coordinated within different learning systems.

## Figures and Tables

**Table 1:** Overview of Sample Size, Variable Coverage, and Mathematics Performance Across Regions.

Country/Region	Sample Size	Variables	Mean Score	Rank	Region
China (B–S–J–Z)	12,058	239	591	1	East Asia
Singapore	6676	404	569	2	East Asia/Southeast Asia
Macao (China)	–	–	558	3	East Asia
Hong Kong (China)	6037	314	551	4	East Asia
Chinese Taipei	–	–	531	5	East Asia
Japan	6109	331	527	6	East Asia
Korea	6650	650	526	7	East Asia
Estonia	5316	509	523	8	Europe
Netherlands	4765	337	519	9	Europe
Poland	5625	585	516	10	Europe
Switzerland	–	–	515	11	Europe
Canada	22,653	362	512	12	North America
Denmark	–	–	509	13	Europe
Slovenia	–	–	509	14	Europe
Belgium	–	–	508	15	Europe

*Note:* “Sample Size” refers to the number of students included after sampling and quality control. “Variables” indicates the number of retained explanatory variables after data preprocessing. For some regions, complete information on sample size or variable coverage is not available in the public dataset and is therefore marked as “–”. Rankings are based on the OECD-reported PISA 2018 mathematics scores.

**Table 2:** Conceptual Framework of the PISA 2018 Student Questionnaire.

Dimension	Sub-Dimension	Representative Constructs/Variables
Student Background	Demographics and SES	Gender, age, parental education, parental occupation, ESCS index, home resources, language at home
Learning Time and Activities	Academic Effort and Support	Homework time, private tutoring, weekly instructional time, study habits
Attitudes, Motivation, and Aspirations	Self-beliefs and Goals	Mathematics self-efficacy, learning motivation, subject interest, perseverance, educational and career expectations
Learning Environment/School Climate	Classroom and School Perceptions	Teacher support, disciplinary climate, teacher–student relations, sense of belonging
Well-being	Psychological and Social Wellness	Life satisfaction, test anxiety, sense of meaning, bullying, physical activity
Reading Habits and Strategies	Reading Frequency and Metacognition	Reading materials, recreational reading, summarizing and retrieval strategies
Digital Literacy and Online Behavior	Digital Skills and Safety	Device use, phishing detection, information verification, digital reading
Global Competence	Global Awareness and Civic Values	Intercultural respect, adaptability, civic engagement, global issues awareness

**Table 3:** Benchmarkd Comparison Across Classification Models (Poland Sample).

Model	Accuracy	Macro F1	Weighted F1
Logistic Regression	0.55	0.24	0.39
Random Forest	0.77	0.57	0.74
CatBoost	0.87	0.83	0.87

Note: Benchmark models were estimated using the Poland sample under the same classification framework after excluding direct mathematics-score variables from the predictor set. Values are reported to provide comparative reference points for predictive differentiation rather than exhaustive optimization results for each classifier.

**Table 4:** Classification Performance of the CatBoost Model Across Regions.

Country/Region	Accuracy	Macro F1	Weighted F1	High F1	Low F1	Medium F1	N
Shanghai	0.73	0.66	0.73	0.74	0.49	0.76	2412
Hong Kong	0.69	0.65	0.68	0.57	0.63	0.75	1208
Japan	0.74	0.64	0.72	0.42	0.72	0.79	1222
Korea	0.86	0.86	0.86	0.87	0.84	0.88	1330
Poland	0.87	0.83	0.87	0.73	0.88	0.89	1125
Estonia	0.76	0.63	0.74	0.34	0.72	0.82	1064
Netherlands	0.83	0.75	0.82	0.53	0.88	0.85	953
Canada	0.87	0.82	0.87	0.69	0.88	0.88	4531

Note: F1 scores represent the harmonic mean of precision and recall. Macro F1 assigns equal weight to each class, whereas Weighted F1 accounts for class proportions. High-, medium-, and low-performing groups were defined based on the distribution of students’ mathematics scores within each regional sample.

**Table 5:** Comparative SHAP Concentration Ratios Across Educational Systems.

Educational System	Top-10 SHAP Contribution Ratio
Shanghai	0.71
Hong Kong	0.69
Japan	0.73
Korea	0.75
Poland	0.52
Estonia	0.49
Netherlands	0.47
Canada	0.45

Note: The concentration ratio represents the cumulative contribution of the top-ranked SHAP predictors relative to total predictor importance within each educational system. Higher values indicate comparatively concentrated predictor organization.

**Table 6:** Cross-Regional Comparison of Structural Pathway Patterns Based on Standardized Coefficients.

Pathways	Shanghai	Hong Kong	Japan	Estonia	Korea	Norway	Canada	Poland	Organizational Tendency
*(1) Contextual and Motivational Organization*									
Educational Expectations → Achievement	0.469 ***	—	—	—	0.167 ***	—	0.122 ***	—	Comparatively Concentrated
Family Background → Achievement	0.392 ***	—	—	—	—	−0.097 ***	0.129 ***	0.150 ***	
Learning Goals → Achievement	0.109 ***	—	—	—	—	—	—	—	
*(2) Psychological Organization*									
Self-beliefs → Achievement	−0.177 ***	—	—	—	—	—	(Indirect)	—	
Anxiety → Achievement	0.022 *	—	—	—	—	—	—	—	
**Psychological Composite** → Achievement	—	—	0.674 ***	0.236	0.167 ***	(Indirect/unstable)	(Indirect)	0.254	
*(3) Behavioral and Environmental Organization*									
Learning Behavior → Achievement	—	0.490 ***	—	0.123	0.105 ***	0.446 ***	0.392 ***	0.266	
Learning Time → Achievement	—	0.170 ***	0.045	0.067	0.024 *	—	—	0.143 ***	
Classroom Practice → Achievement	—	0.231 ***	—	—	—	0.231 ***	0.242 **	—	
Digital Behavior → Achievement	—	—	—	0.090 **	—	(Indirect)	(Indirect/negative)	−0.131	
Effort → Achievement	—	—	−0.010	—	0.122 ***	—	—	—	
**Predominant Organizational Pattern**	Expectation-oriented	Behavior-oriented	Psychology-oriented	Relatively Distributed	Hybrid Coordination	Digitally Mediated	Behaviorally Mediated	Distributed	**Comparative Pattern**
CFI	0.889	0.890	0.925	0.829	0.870	0.851	0.831	0.864	
RMSEA	0.081	0.073	0.071	0.043	0.070	0.082	0.065	0.093	

Note: Standardized coefficients are reported. * *p* < 0.05, ** *p* < 0.01, *** *p* < 0.001. “Indirect” indicates pathways operating primarily through mediation structures. “Indirect/negative” indicates negative indirect associations. “Indirect/unstable” indicates comparatively weak or non-robust indirect patterns. “—” indicates non-significant or excluded pathways. The organizational tendencies reported here are intended as comparative analytical descriptions rather than fixed or universally invariant mechanism categories.

## Data Availability

The data presented in this study are openly available in the OECD PISA database at https://www.oecd.org/pisa/data/ (accessed on 17 May 2026).

## References

[B1-jintelligence-14-00091] Alagumalai S., Buchdahl N. (2021). PISA 2012: Examining the influence of prior knowledge, time-on-task, school-level effects on achievements in mathematical literacy processes—Interpret, employ and formulate. Australian Journal of Education.

[B2-jintelligence-14-00091] Bandura A. (1997). Self-efficacy: The exercise of control.

[B3-jintelligence-14-00091] Bourdieu P., Richardson J. G. (1986). The forms of capital. Handbook of theory and research for the sociology of education.

[B4-jintelligence-14-00091] Breiman L. (2001). Random forests. Machine Learning.

[B5-jintelligence-14-00091] Bryk A. S., Raudenbush S. W. (1992). Hierarchical linear models: Applications and data analysis methods.

[B6-jintelligence-14-00091] Burkhardt H., Pead D., Stacey K. (2024). Learning and teaching for mathematical literacy: Making mathematics useful for everyone.

[B7-jintelligence-14-00091] Chen G. (2017). A study on the evaluation of student learning outcomes and instructional adaptation mechanisms. Educational Research.

[B8-jintelligence-14-00091] Croce K.-A. (2020). Processing the world through mathematical reasoning. Critical perspectives on mathematics learning.

[B9-jintelligence-14-00091] Denischeva L. O., Savintseva N. V., Safuanov I. S., Ushakov A. V., Chugunov V. A., Semenyachenko Y. A. (2021). Peculiarities of formation and assessment of schoolchildren’s mathematical literacy. Science for Education Today.

[B10-jintelligence-14-00091] Eccles J. S., Wigfield A. (2002). Motivational beliefs, values, and goals. Annual Review of Psychology.

[B11-jintelligence-14-00091] Gabriel F., Buckley S., Barthakur A. (2020). The impact of mathematics anxiety on self-regulated learning and mathematical literacy. Australian Journal of Education.

[B12-jintelligence-14-00091] Gao X., Li N. (2024). Applications of machine learning methods in test security. Advances in Psychological Science.

[B13-jintelligence-14-00091] Hanushek E. A. (1979). Conceptual and empirical issues in the estimation of educational production functions. The Journal of Human Resources.

[B14-jintelligence-14-00091] Hattie J. (2009). Visible learning.

[B15-jintelligence-14-00091] Hooper D., Coughlan J., Mullen M. R. (2008). Evaluating model fit: A synthesis of the structural equation modelling literature. The Electronic Journal of Business Research Methods.

[B16-jintelligence-14-00091] Huang L. (2016). An analysis of factors influencing mathematics achievement of first-year high school students in Guiyang. Mathematics Education in Secondary Schools.

[B17-jintelligence-14-00091] Kaiser G., Blömeke S. (2013). Learning from the Eastern and the Western debate: The case of mathematics teacher education. ZDM Mathematics Education.

[B18-jintelligence-14-00091] Kaplan D. (2009). Structural equation modeling: Foundations and extensions.

[B19-jintelligence-14-00091] Lundberg S. M., Lee S.-I. (2017). A unified approach to interpreting model predictions. Advances in neural information processing systems 30.

[B20-jintelligence-14-00091] OECD (2019). Pisa 2018 assessment and analytical framework.

[B21-jintelligence-14-00091] OECD (2020). Pisa 2018 technical report.

[B22-jintelligence-14-00091] Qirom M. S., Turmudi, Juandi D. (2023). Gender difference in mathematical literacy and factor that may affect it. MaPan: Journal of Mathematics and Learning.

[B23-jintelligence-14-00091] Rutkowski L., von Davier M., Rutkowski D. (2014). Handbook of international large-scale assessment: Background, technical issues, and methods of data analysis.

[B24-jintelligence-14-00091] Skwarchuk S.-L., Douglas H., Cahoon A., LeFevre J.-A., Xu C. (2022). Relations between the home learning environment and the literacy and mathematics skills of eight-year-old Canadian children. Education Sciences.

[B25-jintelligence-14-00091] Stacey K. (2011). The PISA view of mathematical literacy in Indonesia. Journal of Mathematics Education.

[B26-jintelligence-14-00091] Stacey K. (2015). The international assessment of mathematical literacy: PISA 2012 framework and items. Selected regular lectures from the 12th international congress on mathematical education.

[B27-jintelligence-14-00091] Steen L. A. (2001). Mathematics and democracy: The case for quantitative literacy.

[B28-jintelligence-14-00091] Tso T.-Y., Lei K.-H. (2018). Design and development of mathematical literacy-oriented subject materials. Journal of Research in Education Sciences.

[B29-jintelligence-14-00091] Wang M.-T., Guo J., Degol J. L. (2020). The role of sociocultural factors in student achievement motivation: A cross-cultural review. Adolescent Research Review.

[B30-jintelligence-14-00091] Xiao H. (2022). Evaluation of student learning outcomes in higher education under the perspective of professional accreditation. Research in Higher Education.

[B31-jintelligence-14-00091] Zhang H. (2018). Individual cognitive and contextual factors affecting Chinese students’ mathematical literacy: A hierarchical linear modeling approach using Program for International Student Assessment (PISA) 2012. Doctoral dissertation.

[B32-jintelligence-14-00091] Zhang Y. (2020). An exploration of factors influencing primary school students’ mathematics achievement. Basic Education Research.

[B33-jintelligence-14-00091] Zhao M., Glewwe P. (2010). What determines basic school attainment in developing countries? Evidence from rural China. Economics of Education Review.

[B34-jintelligence-14-00091] Zhu Y., Leung F. K. S. (2011). Motivation and achievement: Is there an East Asian model?. International Journal of Science and Mathematics Education.

[B35-jintelligence-14-00091] Zimmerman B. J., Boekaerts M., Pintrich P. R., Zeidner M. (2000). Attaining self-regulation: A social cognitive perspective. Handbook of self-regulation.

